# The London University

**Published:** 1898-06-18

**Authors:** 


### The London University.

We are glad to find that the London University Commission Bill passed its second reading in the House of Commons on Tuesday, for although as it stands the Bill is by no means everything that might have been wished, it is a great move in the right direction, and indeed is such a move that if it should be passed without further mutilation, it will at last be possible to arrange something like university teaching in London. We say "at last," for indeed it has only been by dint of much weary waiting that the scheme for giving London a teaching university has arrived even at its present point. For more than twenty years the demand for the establishment of such an institution has received the support of the most eminent scientific men of the day, yet all their efforts have, so far, been frustrated by those already in possession of the field. It is a curious instance of the power of a name and the influence of a mere word, that the present examining body, merely by virtue of the fact that it is called the University of London, should have able for so long to debar Londoners from the possession of a real university.

At the present moment when the future of London as a centre for higher education depends largely upon what happens to this Bill in Committee, it is important to recognise how entirely the opposition to it hinges upon what are maintained to be the rights of the present members of the University, and how entirely the rights of Londoners and the demand for university education go in favour of the Bill. If we may take Mr. Moulton's last letter to the *Times* as expressing the position taken by the opponents of the Bill, we see clearly enough that the education of the rising generation is not what is in their hearts, but rather the maintenance of the value of the hall-mark with which they are already stamped. It is said that the Bill is "an attempt to alter the constitution of the London University against its will," but surely the will of the University has nothing to do with the question. Again, it is urged that the present university has been so successful that the entrance fees paid for its examinations are sufficient to pay all its expenses, and that it has kept its standard of examination high, and we are plaintively asked, "What excuse then can be found for this harsh treatment?" as if the fact of it being a very good, and a commercially successful, examining body in any way touched the question of the wants of London.

No one denies that the University of London has been a success in its own way, but on the other hand, no one can deny the right of London to have a University of its own. Sir Joshua Fitch sums up what Mr. Moulton has to say on the subject as amounting to this: "That the Bill should be rejected because it is believed to be unacceptable to a majority of the present graduates." But even if it were unacceptable to a majority of them, the rights and wants of London should come first. The letter by Professor Sylvanus Thompson, which appears in the *Times* side by side with that of Mr. Moulton, puts the matter perfectly plainly. The present condition of affairs is intolerable; it works very prejudicially upon the colleges, medical schools, and science schools in and around London, and there are only two ways out of the difficulty. One is to create a second and separate teaching university, the other is to reconstitute the present university by adding to the sole function which it is empowered to exercise as an examining board the higher and more direct functions of a university—namely, the duty of organising the means of education, the co-ordination of the colleges and of the teaching, the improvement of the curricula, the providing of facilities for study, the advancement of scholarship, and the encouragement of research.

In every way the second course is the best. The creation of two separate universities has been condemned on all hands, and as regards the present Bill Professor Thompson says that it closes no avenue to any degree that is at present open; it excludes no student at present eligible as a candidate; it opens new avenues to degrees which, by the better organisation and extension of teaching, will be particularly helpful to many poor students to whom university teaching has hitherto been inaccessible; it will give the colleges a real, but not a preponderating, voice upon the Senate, where now they are unrepresented; it will remedy the grievance—if grievance it be—that at present in many subjects both the examiners are London teachers, since it provides that henceforth one at least of the examiners in each examination shall be a person who is not a London teacher. This ought to be enough, and we can only express the hope that the Bill will not be mutilated in Committee. It is the result of twenty years of discussion, and, during late years, of much compromise, and it ought now to become law.

